# Special Issue “Gap Junction Channels and Hemichannels in Health and Disease”

**DOI:** 10.3390/ijms26199264

**Published:** 2025-09-23

**Authors:** Barbara Rijtano, Mario Bortolozzi

**Affiliations:** 1 Veneto Institute of Molecular Medicine (VIMM), 35129 Padua, Italy; 2 Department of Neuroscience, University of Padua, 35129 Padua, Italy; 3 Department of Physics and Astronomy “G. Galilei”, University of Padua, 35131 Padua, Italy

Connexins (Cxs) are a family of tetraspan membrane proteins encoded by 21 genes in humans [1]. First visualized by electron microscopy in 1963 [2], it took more than two decades before a connexin gene (*GJB1*) was cloned [3], and another seven years before its link to Charcot–Marie–Tooth disease type 1X was established [4]. Since then, our understanding of connexins has expanded dramatically. These proteins are now recognized as central players in numerous physiological and pathological processes, owing to their diverse biological functions. Depending on the cellular and tissue context, connexins contribute to cell–cell communication and adhesion, extracellular signalling and vesicle release, mitochondrial biogenesis, transcriptional regulation, and nanotube tunnelling [5]. Their functions depend on the ability of connexin isoforms to assemble into hexameric channels known as connexons or hemichannels (HCs). Within the plasma membrane, two HCs from adjacent cells can dock end-to-end to form a gap junction channel (GJC), enabling direct cytosolic exchange of ions and small metabolites [6]. Understanding the contribution of distinct connexin channels to physiology and disease is therefore critical, with major implications for the development of novel therapeutic strategies. Through fourteen contributions published in the two Special Issues of the *IJMS* Biochemistry Section, *Gap Junction Channels and Hemichannels in Health and Disease* (1st and 2nd editions), we aim to present new insights and perspectives to advance the field and address these fundamental questions.

Emerging evidence suggests that neuronal and glial communication via GJCs amplifies neuroinflammation and neurodegeneration in Parkinson’s and Alzheimer’s diseases. Contribution 1 of Pechlivanidou et al. presented the first study investigating Alzheimer’s pathology in the spinal cord rather than the brain, using the 5xFAD mouse model. They reported increased immunoreactivity of oligodendroglial Cx47 GJCs on cell bodies, along with elevated expression of Cx43 and Cx30 around amyloid-β deposits. This study broadens our understanding of the cellular contributors to Alzheimer’s progression and suggests that connexin-mediated chronic inflammation may represent a promising therapeutic target. Underscoring the complex tissue-specific consequences of connexin modulation, Contribution 2 of Li et al. showed in mouse models that selective impairment of Cx43 HCs preserves bone mass during ageing but compromises skeletal muscle function.

Connexin function is tightly regulated at multiple levels. At the post-transcriptional stage, microRNAs such as miR-206 and miR-133a suppress connexin translation, while lncRNAs and circRNAs act as sponges to promote expression (Contribution 3). At the protein level, connexin trafficking, turnover, and recycling are mainly controlled by clathrin-mediated endocytosis, although isoform-specific variations in internalization mechanisms exist (Contribution 4). Dysregulation of these processes can amplify inflammatory responses, particularly in the central nervous system, through aberrant ATP release and mediator signalling (Contribution 5).

The biophysical regulation of GJs and HCs is a key determinant of their physiological and pathological functions. Voltage, pH, and Ca^2+^ ions finely tune channel gating. Peracchia (Contributions 6–7) proposed a model in which a fast transjunctional voltage sensor and a slower Ca^2+^-dependent “cork” mechanism mediated by calmodulin (CaM) cooperate to control GJC opening and closure. Contribution 8 of Tran et al. expanded this model, showing that sub-micromolar cytosolic Ca^2+^ variations induce a conformational stretch in the intracellular loop region of GJCs, leading to CaM-mediated pore closure. Bayraktar et al. (Contribution 9) highlighted the presence of a dual Ca^2+^-dependent gate in HCs: intracellular Ca^2+^ triggers HC opening that allows release of extracellular bursts of messenger molecules, whereas extracellular Ca^2+^ stabilizes channel closure. A comprehensive molecular framework is still needed to fully understand HC gating and to guide the design of isoform-selective modulators.

From a therapeutic perspective, connexins offer both challenges and opportunities. Broad-spectrum blockers such as carbenoxolone and octanol inhibit connexin-mediated communication but lack isoform specificity, limiting their clinical potential [7]. More selective approaches are emerging: peptide-based modulators such as Gap19 (targeting Cx43 HCs) and ^10^Panx1 (targeting Panx1 HCs) show promise, though issues of cross-reactivity and precise mechanisms remain under investigation (Contribution 10). Della Morte et al. reported in Contribution 11 that pro-inflammatory cytokines (IL-1β/TNF-α) upregulate Cx43 and promote HC opening in synovial fibroblasts, triggering ATP-dependent IL-6/IL-8 release, a process reversible by TAT-Gap19 or Cx43 siRNA. These findings point to HCs as potential therapeutic targets in rheumatoid arthritis. Conversely, enhancing connexin-mediated communication may be beneficial in other settings. Contribution 12 of Buchberger et al. demonstrated that Cx43 GJCs between endothelial progenitors and mature cells are essential for angiogenesis, suggesting strategies for ischemic tissue repair. Moreover, Peracchia described in Contribution 13 how common anesthetics modulate GJC gating, with implications for both clinical safety and mechanistic insights into connexin regulation. Environmental factors also influence connexin function. Contribution 14 of Yin et al. showed that microwave radiation alters Cx43 expression and localization in iPSC-derived cardiomyocytes, consistent with prior observations of electrophysiological disturbances and mitochondrial dysfunction in animal models [8].

In summary, connexins and their HCs and GJCs are central to tissue homeostasis and disease. The key challenge will be to identify which connexin isoforms and functions should be targeted in specific pathological contexts, including neurodegeneration, inflammation, bone remodelling, and cardiovascular function. The studies presented in these two Special Issues expand our understanding of connexin biology and highlight new therapeutic opportunities. We hope these contributions will inspire further investigations that lay the foundation for precision medicine approaches to connexin-targeted therapies.
